# Metabolic Characterization of Anterior Mediastinal Masses by ^18^F-FDG PET/CT

**DOI:** 10.4274/mirt.galenos.2020.05657

**Published:** 2020-10-19

**Authors:** Zehra Pınar Koç, Pınar Pelin Özcan, Erhan Ayan, Rabia Bozdoğan Arpacı

**Affiliations:** 1 Mersin University Faculty of Medicine, Department of Nuclear Medicine, Mersin, Turkey; 2 Mersin University Faculty of Medicine, Department of Thoracic Surgery, Mersin, Turkey; 3 Mersin University Faculty of Medicine, Department of Pathology, Mersin, Turkey

**Keywords:** Anterior mediastinum, mass, 18F-FDG, PET/CT

## Abstract

**Objectives:**

To evaluate the role of ^18^F-fluorodeoxyglucose (FDG) positron emission tomography/computed tomography (PET/CT) for the diagnosis of anterior mediastinal masses.

**Methods:**

The oncological ^18^F-FDG PET/CT images of 41 patients (17 women, 24 men; age: 16-83 years, mean age: 50.5±19.5 years) who attended the nuclear medicine department between November 2016 and September 2017 were retrospectively evaluated for the metabolic characterization of their anterior mediastinal masses.

**Results:**

Based on our results, the lesions of 4 patients were benign [maximum standard uptake value (SUV_max_) <3] and that of 2 patients were non-tumoral (i.e., tuberculosis and sarcoidosis). The mean dimensions and the SUV_max_ levels of the malignant lesions were 6.4±3.7 cm and 11.9±9.6, respectively. The pathological results for the malign tumors were thymus tumors (n=8), lymphoma (n=8), lung cancer (n=11), carcinoid metastasis (n=2), thyroid carcinoma (n=2), germ cell carcinoma (n=1), schwannoma (n=1), and sarcoma (n=1). The degree of ^18^F-FDG accumulation could precisely identify the malign and benign tumors.

**Conclusion:**

Thus, contrary to the known causes, it is possible that anterior mediastinal masses originate from structures other than the anterior mediastinal structures. In this study, the lymphoma and lung carcinoma pathology were more frequent than thymic lesions.

## Abstract (tr)

**Amaç:**

Bu çalışmada anterior mediastinal kitlelerin karakterizasyonunda ^18^F-florodeoksiglukoz (FDG) pozitron emisyon tomografi/bilgisayarlı tomografinin (PET/BT) rolü değerlendirilecektir.

**Yöntem:**

Anterior mediastinal kitle tanısıyla nükleer tıp bölümüne Kasım 2016-Eylül 2017 tarihleri arasında metabolik karakterizasyon amacıyla başvuran hastaların onkolojik ^18^F-FDG PET/BT görüntüleri geriye dönük olarak değerlendirildi. Kırk bir hasta (17 kadın, 24 erkek; 16-83, ortalama yaş: 50,5±19,5) çalışmaya dahil edildi.

**Bulgular:**

Çalışmaya dahil edilen hastalardan iki hastanın lezyonu benign olarak tanımlandı [maksimum standart alım değeri (SUV_maks_) <3] ve ikisinin patolojisi tümör dışı lezyonlardı (tüberküloz ve sarkoidoz). Malignite tanısı konulan hastaların kitlelerinin ortalama boyutu ve SUV_maks_ değerleri sırasıyla; 6,4±3,7 cm ve 11,9±9,6 idi. Malign tümörlerin patolojik tanıları; timik tumörler (n=8), lenfoma (n=8), akciğer kanseri (n=11), karsinoid metastazı (n=2), tiroid karsinomu (n=2), germ hücreli tümör (n=1), schwannoma (n=1) ve sarkom (n=1) idi.

**Sonuç:**

Bilinen nedenlerin aksine anterior mediastinal kitlelerin patoloji sonuçları anterior mediastinal yapıların tümörlerinin veya karsinomlarının dışındaki patolojilerden kaynaklanabilir. Bu seride timik tümörlere göre lenfoma ve akciğer karsinomu daha sık rastlanan patolojilerdi.

## Introduction

Several causes of anterior mediastinal masses, either benign or malignant, have been reported. In fact, it has been reported that anatomic structures in the anterior mediastinum either enlarge or become malignant or metastasize from another tumor. Several studies have also evaluated thymus enlargement as a differential diagnosis of anterior mediastinal masses (1). In addition, numerous cases have been reported related to the origin of anterior mediastinal masses in the literature (1). The other reasons of development of anterior mediastinal mass are benign enlargement of anatomic structures involving or invading the mediastinum (2,3).

It has been previously documented that the ^18^F-fluorodeoxyglucose (FDG) positron emission tomography/computed tomography (PET/CT) is an accurate modality in the staging and restaging of anterior mediastinal tumors. In fact, a recent study suggested that the diagnostic power of ^18^F-FDG PET/CT for anterior mediastinal mass is high, but its negative predictive value is higher (4). Another small series of study determined the cut-off level for the determination of malignancy in anterior mediastinal mass (5). The present study aimed to evaluate the pathological outcomes of adult patients presenting with anterior mediastinal mass in conjunction with the ^18^F-FDG PET/CT findings.

## Materials and Methods

### Patients

The following were the patient inclusion criteria: age of 16-85 years and presenting with anterior mediastinal mass without histopathological diagnosis.

Informed consents were obtained from the patients for conducting PET/CT examinations.

The following were the patient exclusion criteria: pregnancy and lactation, age <16 or >85 years, presenting with another malign tumor elsewhere, and those with contraindication for PET/CT examinations.

The study was approved by the Local Ethics Committee and conducted according to the revised Helsinki Declaration, 2010. Mersin University Rectorate Clinical Research Ethics Committee (date: 05/10/2017, no: 2017/285).

The PET/CT images of 41 patients (17 women, 24 men, age: 16-83 years, mean age: 50.5±19.5 years) who were referred to the nuclear medicine department with the diagnosis of anterior mediastinal mass by a previous CT examination conducted between November 2016 and September 2017 were obtained. The data were retrospectively evaluated by 2 experienced nuclear medicine physicians without any knowledge of the final diagnosis of the patients. The PET/CT study was not performed for patients with anamnesis of pregnancy and lactation and for those with contraindication for the examination. In addition, we did not prefer pediatric patients with interfering problems that involved the anterior mediastinum frequently (such as physiological thymus activity) and elder patients (age: >85 years) because they probably could not be operated.

### PET/CT Examinations

The patients were prepared for the examination by ensuring at least 6 h of fasting and decreased physical activities since at least 24 h prior to the examination. The patients were first injected with the radiopharmaceutical agent [mean 370 MBq (10 mCi), according to the body weight] via the venous line 60 min before the imaging. Imaging was performed by using a PET/CT scanner (discovery PET/CT 610; GE, US) with a low-dose CT scan (130 kV, 50 mAs, 1.5 pitch, 5-mm thickness, 70-cm field of view) for attenuation correction without intravenous contrast administration via oral contrast administration from the skull base to the upper thigh region with the acquisition time of 3 min/bed position and the matrix size of 256x256. Attenuation-corrected PET images were then reconstructed by using an iterative reconstruction algorithm, VUE point HD with 3 iterations and 32 subsets.

### Diagnostic Criteria

The images were evaluated with respect to the metabolic characteristics of the anterior mediastinal lesions [maximum standard uptake value (SUV_max_)] levels obtained from the workstation (Mac iOs, Osirix MD programme). The SUV_max_ levels were retrieved by the circular region of interest covering the most active portions of the lesions in addition to the CT characteristics of the lesions and the dissemination to other structures (metastatic dissemination, lymphadenopathies elsewhere in the body, and other possible malignant primary sites). The anterior mediastinal mass lesions were determined to be benign in case of a single site with low uptake of ^18^F-FDG (SUV_max_ <3).

### Interventions and Histopathological Analysis

Surgical procedures were decided with reference to the PET/CT imaging and suspected malignancy. The types of surgical procedures conducted (such as thoracotomy, minithoracotomy sternotomy procedures, or biopsy) for each patient are summarized in Table 1. The final pathological outcomes obtained from the specimens of surgery including hematoxylene and eosine or immunohistochemistry (in case it was necessary) staining procedures were analyzed by an experienced pathology physician, and the results of the PET/CT and pathology were compared.

### Statistical Analysis

The statistical analysis was performed by using a package program (MedCalc^®^v10.3.0). The receiver operating curve (ROC) analysis was performed in order to determine the power of the SUV_max_ parameter to differentiate between the benign and malignant lesions.

## Results

**Figure 1:**
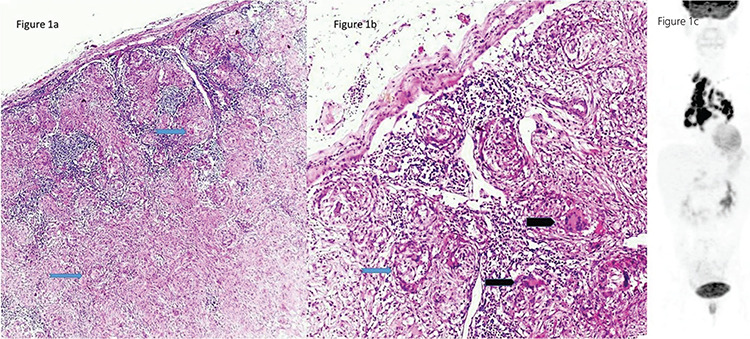
39-year-old woman with pathological diagnosis of sarcoidosis was evaluated. Her histological sections revealed well-defined, small, nonnecrotizing granulomas composed of epithelioid cells with scattered (a) Langhans giant cells in the lymph node. Necrosis was absent. Granuloma was noted at higher power. Non-necrotizing granulomas composed of epithelioid cells with scattered Langhans giant cells (H&E, x40). (b) Langhans giant cells can be observed in the middle area. These cells are presenting with mediastinal multiple lymph nodes with significantly increased ^18^F-FDG affinity (H&E, x200), as demonstrated by the (c) multiple intensity projection image of ^18^F-FDG PET/CT H&E: Haemotoxylin and eosin, ^18^F-FDG: ^18^F-fluorodeoxyglucose

**Figure 2:**
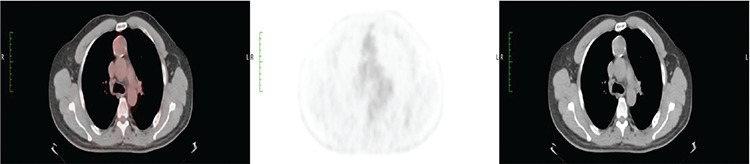
The ^18^F-FDG PET/CT images of a 56-year-old man with anterior mediastinal mass in the transaxial projection of fusion, PET, and CT with low ^18^F-FDG uptake who was out of the follow-up program without any pathological outcome ^18^F-FDG: ^18^F-fluorodeoxyglucose, PET/CT: Positron emission tomography/computed tomography

**Figure 3:**
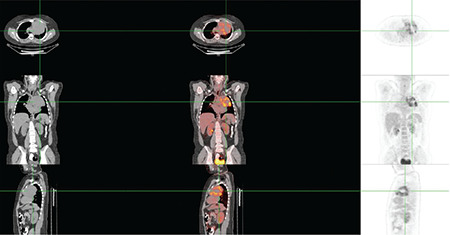
Heterogeneous uptake with increased ^18^F-FDG accumulation in large anterior mediastinal mass, which was immature teratoma, as shown by the transaxial, coronal, and sagittal plane CT, fusion, and PET images ^18^F-FDG: ^18^F-fluorodeoxyglucose, PET: Positron emission tomography, CT: Computed tomography

**Figure 4:**
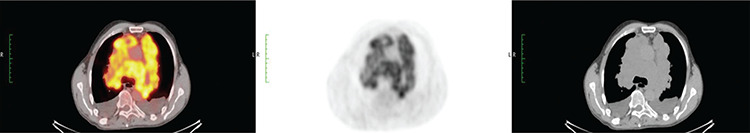
Transaxial fusion, PET, and CT images of anterior mediastinal conglomerated hypermetabolic mass lesion diagnosed as small cell lung carcinoma PET: Positron emission tomography, CT: Computed tomography

Of the 41 study participants, 37 underwent different surgeries based on their imaging findings (Table 1). The pathological results including those of 2 patients with the diagnosis of granulomatous diseases (Figure 1) are listed in Table 1. A total of 4 patients were considered to be benign based on their PET/CT imaging, these patients did not undergo any surgical procedure and were also out of the follow-up program (Figure 2). In this series, 10 patients were diagnosed with lymphoma, while 2 were diagnosed with neuroendocrine tumor metastases with a relatively low ^18^F-FDG uptake. One of the patients had immature teratoma with a significantly high metabolic activity (Figure 3). The patient diagnosed with differentiated thyroid carcinoma also showed high ^18^F-FDG affinity; however, those with medullary thyroid carcinoma showed a relatively low uptake. Unexpected results, such as lung carcinoma, were recorded in the study group as well (Figure 4).

The distribution of the pathological results and the SUV_max_ levels of the lesions are summarized in Table 1. The ^18^F-FDG avid lesions outside of the anterior mediastinum of the patients are listed under Table 1. Thirteen patients underwent follow-up ^18^F-FDG PET/CT imaging (mean 7.4±5.2 month), while 3 patients died within 1 month of PET/CT examination during the disease course. The progression of the disease was noted in 4 patients, with partial response in 2 and complete metabolic response in 5. One patient was diagnosed with interfering infection and 2 with recurrent mediastinal lesion.

**Figure 5:**
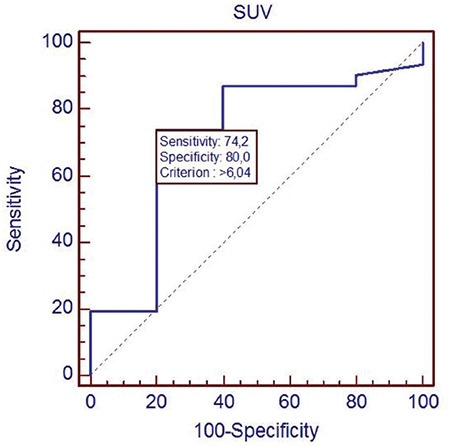
ROC analysis of sensitivity and specificity of the ^18^F-FDG PET/CT according to the SUV_max_ cut-off value (6.04). Graphical demonstration of ROC curves ROC: Receiver operating curve, ^18^F-FDG: ^18^F-fluorodeoxyglucose, PET/CT: Positron emission tomography/computed tomography, SUV_max_: Maximum standard uptake value

The cut-off SUV_max_ level during the determination of malignant and benign tumors was 6.04 as per the ROC curves, and, with this cut-off value, the sensitivity and specificity of the diagnostic modality was 74% and 80%, respectively (Figure 5). The SUV_max_ level was found to be significantly (p=0.05) successful in the determination of malignant lesions, while this level in patients with thymoma was 3-5.95. The SUV_max_ levels of thymic carcinoma was significantly higher than those of thymoma lesions (3-19,39).

## Discussion

The benign anterior mediastinal masses may be benign metastasizing leiomyoma, inflammatory endobronchial pseudotumor, physiological thymus activity, or rebound thymus activity (1). The benign metastasizing leiomyoma is a rare tumor of the middle-age women occurring years after hysterectomy (6). Inflammatory pseudotumor is a benign lesion of children or young adults that may be associated with trauma, paraneoplastic syndrome, or inflammatory reactions (7). It is therefore important to discriminate the thymus uptake or thymic rebound from the pathological uptake in PET/CT among young people and children (1). Unfortunately, patients with benign lesions in this case series were also out of the follow-up and did not want to undergo an operation.

Although some pitfalls and false-positive results are associated with the ^18^F-FDG uptake of the surrounding tissues (8), the ^18^F-FDG PET/CT remains the most important modality in the preoperative evaluation of anterior mediastinal lesions. In this case series, only a limited number of patients showed false-positive results, including sarcoidosis (Figure 1) and tuberculosis. Granulomatous infections frequently interfere with malignant tumor metastasis or primary tumors, especially for PET/CT examinations in endemic countries and provides false-positive results. However, the ratio of false-positive results of patients with granulomatous diseases was found to be in an acceptable range in this study.

The most common tumors of the anterior mediastinum are thymic tumors “thymoma” in adults, and previous studies have demonstrated a close correlation between the World Health Organization and Mosaka stage of the thymic tumors and SUV_max_ levels (9). This study group included only 3 patients with thymoma, which is rarer than expected, while the SUV_max_ levels of these patients were in an acceptable range. The thymic carcinoma group showed higher SUV_max_ levels, as expected, than the thymoma group. CT or magnetic resonance showed extension and invasion into the adjacent mediastinal structures of the thymic tumors (9). The ^18^F-FDG PET/CT may differentiate the subgroup of thymic epithelial tumors such as thymoma, thymic carcinoma, and carcinoid tumors and accurately stage these tumors (10). Complete surgical removal of the thymic tumors with the involved adjacent structures is hence the popular treatment modality (11). A special subgroup of thymic tumors is the cystic thymus tumor that is characterized by multiple cysts (12). The ^18^F-FDG PET/CT imaging may demonstrate an increased uptake in the cystic thymomas related to the septum or margins of the tumor (12). The SUV_max_ cut-off level for malignant and benign thymomas have not been determined, although the previous reports accepted the 4.5-6.3 levels (13). The SUV_max_ cut-off value for the determination of the benign and malignant lesions by ^18^F-FDG PET/CT was 6.04 in this study by the ROC analysis. We noted that the diagnostic sensitivity and specificity of the test was acceptably high with this determined cut-off level.

Another problem with the anterior mediastinum is the physiological uptake related to thymus and a phenomenon called the “thymic rebound” that occurs especially in the pediatric age. Thymic rebound refers to the thymus regression during and enlargement after the completion of chemotherapy (1). A previous related study concluded that the SUV_max_ cut-off level of 3.1 may accurately differentiate thymic rebound from lymphoma recurrence (1). Another study about benign and malignant anterior mediastinal mass indicated the cut-off level of 3 (5). Other malignant tumors of anterior mediastinum include lymphoproliferative diseases such as lymphoma, neuroendocrine tumors, and mesenchymal tumors. The most important change in the patients’ management was observed in the lymphoma group since the biopsy sites were altered due to the PET results. In a case series on patients with lymphoma, it was suggested that mild ^18^F-FDG accumulating lesions in the anterior mediastinal region may indicate benign lesions like thymic hyperplasia even in lymphoma patients (14). However, in the case of the presence of diagnostic criteria indicating malignancy in lymphoma patients, secondary malignant tumors of the anterior mediastinum have been indicated in previous case reports (15).

### Study Limitations

The study limitations include the retrospective design that limits the patient selection. In addition, relatively small number of patients could be included since the main subject of the study considered specific patient population.

## Conclusion

This study thus demonstrated that adult patients, especially, may have several unexpected other primary malignancies, especially lung cancer (30%). No study has so far reported the pathological outcomes of anterior mediastinal mass in comparison with the ^18^F-FDG PET/CT imaging results. We demonstrated that ^18^F-FDG PET/CT is an essential imaging modality for the characterization of anterior mediastinal mass. This modality may change the patients’ management by determines other possible biopsy sites.

## Figures and Tables

**Table 1:**
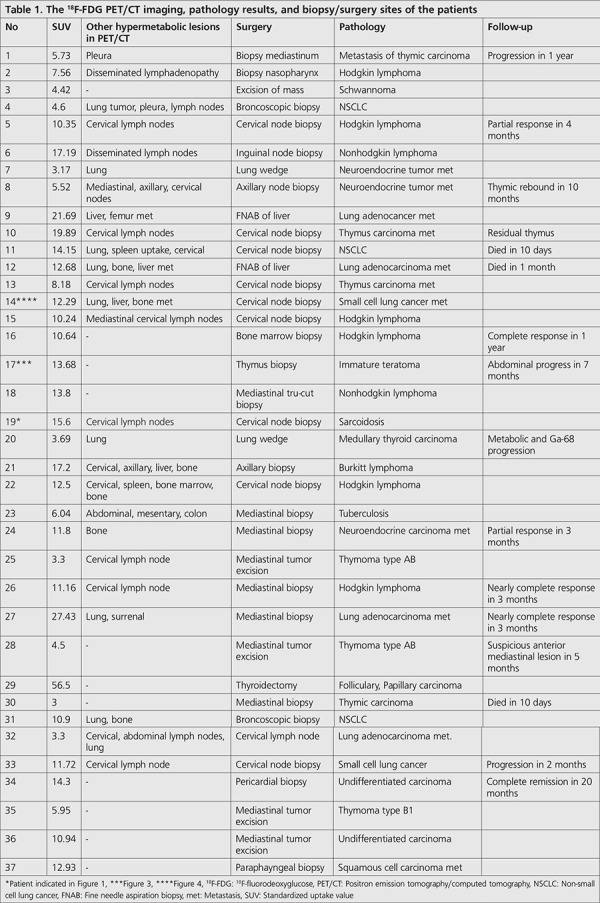
The ^18^F-FDG PET/CT imaging, pathology results, and biopsy/surgery sites of the patients
